# Driving Human Motor Cortical Oscillations Leads to Behaviorally Relevant Changes in Local GABA_A_ Inhibition: A tACS-TMS Study

**DOI:** 10.1523/JNEUROSCI.0098-17.2017

**Published:** 2017-04-26

**Authors:** Magdalena Nowak, Emily Hinson, Freek van Ede, Alek Pogosyan, Andrea Guerra, Andrew Quinn, Peter Brown, Charlotte J. Stagg

**Affiliations:** ^1^Oxford Centre for fMRI of the Brain, Nuffield Department of Clinical Neurosciences, and; ^2^Medical Research Council Brain Network Dynamics Unit and Nuffield Department of Clinical Neurosciences, University of Oxford, Oxford OX3 9DU, United Kingdom,; ^3^Oxford Centre for Human Brain Activity, Department of Psychiatry, University of Oxford, Oxford OX3 7JX, United Kingdom,; ^4^Unit of Neurology, Neurophysiology, Neurobiology, Department of Medicine, University Campus Bio-Medico, 00128 Rome, Italy, and; ^5^Department of Neurology and Psychiatry, Sapienza University of Rome, 00185 Rome, Italy

**Keywords:** concurrent tACS and TMS, GABA inhibition, gamma oscillations, motor excitability, motor learning, tACS

## Abstract

Beta and gamma oscillations are the dominant oscillatory activity in the human motor cortex (M1). However, their physiological basis and precise functional significance remain poorly understood. Here, we used transcranial magnetic stimulation (TMS) to examine the physiological basis and behavioral relevance of driving beta and gamma oscillatory activity in the human M1 using transcranial alternating current stimulation (tACS). tACS was applied using a sham-controlled crossover design at individualized intensity for 20 min and TMS was performed at rest (before, during, and after tACS) and during movement preparation (before and after tACS). We demonstrated that driving gamma frequency oscillations using tACS led to a significant, duration-dependent decrease in local resting-state GABA_A_ inhibition, as quantified by short interval intracortical inhibition. The magnitude of this effect was positively correlated with the magnitude of GABA_A_ decrease during movement preparation, when gamma activity in motor circuitry is known to increase. In addition, gamma tACS-induced change in GABA_A_ inhibition was closely related to performance in a motor learning task such that subjects who demonstrated a greater increase in GABA_A_ inhibition also showed faster short-term learning. The findings presented here contribute to our understanding of the neurophysiological basis of motor rhythms and suggest that tACS may have similar physiological effects to endogenously driven local oscillatory activity. Moreover, the ability to modulate local interneuronal circuits by tACS in a behaviorally relevant manner provides a basis for tACS as a putative therapeutic intervention.

**SIGNIFICANCE STATEMENT** Gamma oscillations have a vital role in motor control. Using a combined tACS-TMS approach, we demonstrate that driving gamma frequency oscillations modulates GABA_A_ inhibition in the human motor cortex. Moreover, there is a clear relationship between the change in magnitude of GABA_A_ inhibition induced by tACS and the magnitude of GABA_A_ inhibition observed during task-related synchronization of oscillations in inhibitory interneuronal circuits, supporting the hypothesis that tACS engages endogenous oscillatory circuits. We also show that an individual's physiological response to tACS is closely related to their ability to learn a motor task. These findings contribute to our understanding of the neurophysiological basis of motor rhythms and their behavioral relevance and offer the possibility of developing tACS as a therapeutic tool.

## Introduction

Over the past decades, there has been an increasing understanding of the importance of oscillatory neural activity in underpinning behavior. In the motor domain, synchronized oscillations in the beta (15–30 Hz) and higher-gamma (60–90 Hz) frequency bands are known to be of particular importance. Specifically, beta band oscillations are the dominant oscillatory activity in the primary motor cortex (M1) at rest (Murthy and Fetz, 1992; Salmelin and Hari, 1994) and a reduction in beta oscillatory power is routinely observed immediately before and during transient voluntary movements (movement-related beta desynchronization [MRBD]; Baker et al., 1997; Pfurtscheller and Lopes da Silva, 1999; Hall et al., 2011). Conversely, an increase in gamma power occurs shortly before movement onset and during movement execution (movement-related gamma synchronization [MRGS]) and has been suggested to reflect the initial activation of primary motor neurons subserving movement (Crone et al., 1998; Pfurtscheller and Lopes da Silva, 1999; Pfurtscheller et al., 2003; Cheyne et al., 2008; Muthukumaraswamy, 2010; Cheyne, 2013; Gaetz et al., 2013). Recently, it has been shown in humans that driving higher-gamma oscillations facilitates, and driving beta oscillations inhibits, motor performance (Pogosyan et al., 2009; Joundi et al., 2012), thus demonstrating a causal role of these brain rhythms in motor control.

At a mechanistic level, a growing body of research supports a link between oscillatory activity in both the beta and gamma bands and the balance of excitation and inhibition within reciprocally connected networks of inhibitory GABAergic interneurons and excitatory glutamatergic pyramidal cells within M1 (Whittington et al., 1995; Yamawaki et al., 2008; Atallah and Scanziani, 2009; Gaetz et al., 2011; Hall et al., 2011; Buzsáki and Wang, 2012; Guerra et al., 2016). In humans, however, more research is needed to advance our understanding of the physiological basis underlying these motor rhythms and their behavioral relevance.

Transcranial alternating current stimulation (tACS) is a noninvasive tool that allows the frequency-specific modulation of neural oscillations (Zaehle et al., 2010; Feurra et al., 2011; Ali et al., 2013) and has been valuable for demonstrating the causal nature of the oscillation–behavior relationship (Pogosyan et al., 2009; Thut et al., 2012; Helfrich et al., 2014). Mounting evidence suggests that tACS modulates intrinsic brain oscillations via direct entrainment (Zaehle et al., 2010; Ali et al., 2013; Herrmann et al., 2013; Helfrich et al., 2014; Fröhlich, 2015; Witkowski et al., 2016), so its effect is most pronounced when stimulation frequency matches the natural frequency of the stimulated neuronal elements (Zaehle et al., 2010; Thut et al., 2011; Ali et al., 2013; Reato et al., 2013). tACS therefore has the potential to be used to study local oscillatory activity *in vivo*.

Here, we used transcranial magnetic stimulation (TMS) to evaluate the neurophysiological basis of driving oscillatory activity *in vivo* using tACS. Specifically, we examined the effects of beta and gamma frequency tACS applied over the M1 for 20 min. The effects of tACS on resting physiological measures were examined and, due to the well recognized patterns of changes in beta and gamma frequency power during movement preparation, the after-effects of tACS on pre-movement physiological measures were also evaluated. Moreover, given the converging evidence demonstrating the critical role of GABA_A_ inhibition in both motor cortical oscillations and learning (Gaetz et al., 2011; Hall et al., 2011; Stagg et al., 2011; Pollok et al., 2014), we addressed the question of whether the magnitude of tACS-induced changes in GABA_A_ activity could be related to individual differences in motor learning. We hypothesized that beta tACS would increase, and gamma tACS decrease, GABA_A_ inhibition, as assessed via short interval intracortical inhibition (SICI) and, further, that these changes would be related to the degree to which an individual could learn a motor task.

## Materials and Methods

### 

#### Participants

Twenty healthy subjects (age 24.9 years, range: 21–30 years; 9 male) gave their informed consent to participate in the study in accordance with Central University Research Ethics Committee approval (University of Oxford; MSD-IDREC-C2–2014-026). All subjects were right-handed as assessed by the Edinburgh Handedness Inventory (Oldfield, 1971) and had no history of neurological or psychiatric disorders; no metal implants, and reported no other contraindications to tACS, TMS, or magnetoencephalography (MEG).

#### Experimental design and procedure

**Figure 1.**
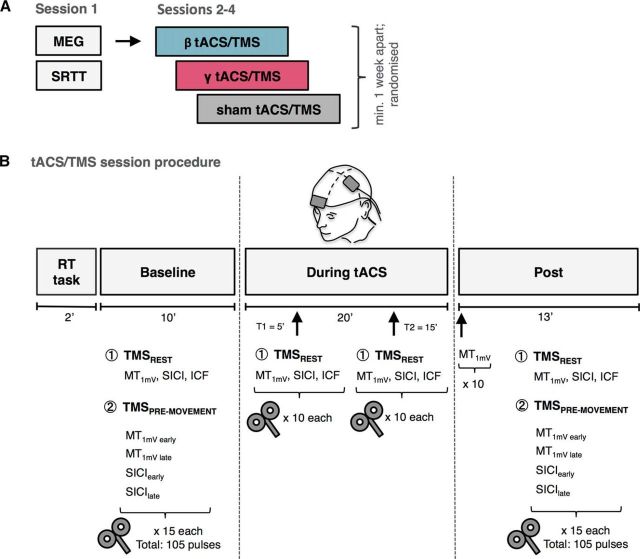
***A***, Study outline. ***B***, Summary of tACS/TMS session procedure. Baseline and post measurements consisted of 15 blocks of 7 TMS pulses: 3 at rest (MT_1mV_, SICI, ICF) and 4 during pre-movement period (MT_1mV early_, MT_1mV late_, SICI_early_, SICI_late_). TMS measurements online to tACS consisted of 10 blocks of 3 pulses: MT_1mV_, SICI, ICF, performed at rest at two time points (T1 = 5 min, T2 = 15 min). SRTT, Serial reaction time task.

Subjects participated in 4 experimental sessions separated by at least 1 week and performed at approximately the same time of the day for each subject (Fig. 1*A*). In Session 1, all subjects had a MEG measurement to determine peak beta and gamma frequency within the left M1 for subsequent tACS sessions. After the MEG recording, participants performed an explicit sequence learning task. In the remaining sessions (Sessions 2–4), subjects received tACS over the left M1 at beta frequency (individual beta frequency [IBF] or 20 Hz), gamma frequency (75 Hz; see below), or sham stimulation, with the order of sessions counterbalanced across subjects. At the start of each tACS session, participants performed a visually cued response time (RT) task.

Measures of corticospinal excitability, intracortical facilitation, and inhibition in left M1 were acquired during eyes open at rest (in the absence of a task) before (baseline), during (online), and after tACS (post) using TMS (these are referred to as resting-state measures). In addition, in conjunction with a RT task performed with the right hand, corticospinal excitability and intracortical inhibition were investigated during the pre-movement period before and after tACS (Fig. 1*B*).

All subjects undertook MEG recording and completed the explicit sequence learning task as well as the beta tACS and sham sessions. Due to scheduling constraints, 18 subjects completed the gamma tACS session. Data from one beta and one gamma tACS session were excluded due to difficulties with TMS data acquisition.

#### MEG

The primary goal of the MEG recording was to acquire resting-state and motor task-related data for the purpose of identification of the peak beta and gamma frequency, respectively, for subsequent tACS sessions.

##### MEG data acquisition.

MEG data were acquired with a whole-head 306-channel Elekta Neuromag system (204 planar gradiometers, 102 magnetometers). Concurrent surface electromyography (EMG) of the right extensor digitorum communis and first dorsal interosseous (FDI) muscle were recorded using bipolar surface electrodes. Both MEG and EMG data were sampled at 1000 Hz with a band-pass filter of 0.03–330 Hz and stored for offline analysis. Head position was continuously monitored with respect to the MEG sensors using four head-position (HPI) coils. The locations of HPI coils and of three anatomical fiducials (the nasion and two preauricular points) were digitized using a 3D tracking system (Polhemus, EastTrach 3D) to define the subject-specific Cartesian head coordinate system. In addition, vertical and horizontal electro-oculogram electrodes were used to allow for detection and removal of eye-blink artifacts.

MEG data were acquired during a single session consisting of resting-state (8 min; eyes open) and a Go/NoGo task. In the Go/NoGo task, a blue circle cue, presented for 200 ms, instructed participants to prepare for an abduction of the index finger of their right hand. The cue was then replaced by a fixation cross for 1000 ms (cue–stimulus interval). A subsequent visual stimulus presented for 200 ms (colored circle: green for Go or red for NoGo) indicated whether they should perform (Go) or withhold (NoGo) the prepared response. Participants were instructed to respond as quickly as possible on the Go trials. The stimulus was then replaced by a fixation cross for a duration that varied randomly between 2000 and 4000 ms (intertrial interval). The task consisted of a total of 70 trials and lasted ∼5 min. NoGo trials (20% of all trials) were introduced to encourage participants' attention to the task.

Stimuli were generated using the MATLAB Psychophysics Toolbox version 3.0 package (Brainard, 1997) and back-projected (Panasonic DLP Projector, PT D7700E) onto a screen at a viewing distance of 120 cm with a spatial resolution of 1024 by 768 pixels and a refresh rate of 60 Hz.

##### MEG analysis.

MEG data were analyzed using the OHBA Software Library, Fieldtrip (Oostenveld et al., 2011), and Elekta software.

##### MEG data preprocessing.

**Figure 2.**
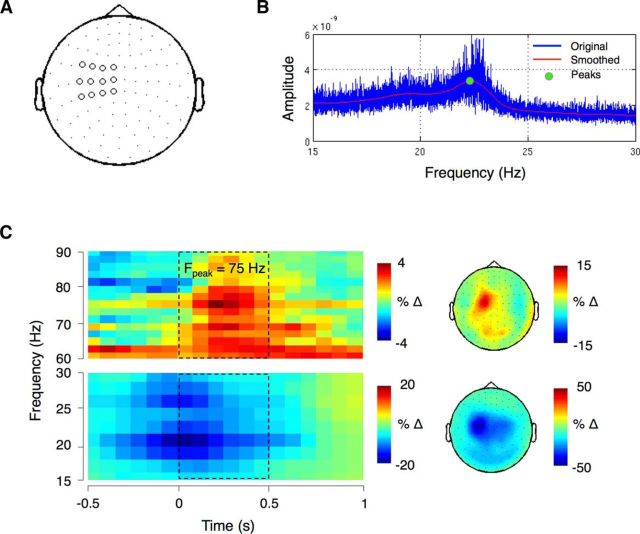
***A***, Sensor selection for left sensorimotor cortex. ***B***, Amplitude spectrum of one example subject who showed a clear beta peak. ***C***, Left, Grand mean time–frequency representation time locked to the onset of EMG activity (at 0 s) for all subjects. The dashed rectangles denote approximate movement period (0–0.5 s). *F*_peak_ indicates the mean peak frequency. Right, Topography in the higher gamma band (60–90 Hz; top) and beta band (15–30 Hz; bottom) over the time–frequency window marked with dashed rectangles on the left. Power is expressed as a percentage change from a 1 s prestimulus baseline as follows: (power − baseline/baseline) * 100.

The raw MEG data were first inspected visually to remove channels with high levels of noise and then the temporal extension of Maxfilter signal space separation method was applied, together with the detection of statistically bad channels and head movement compensation (Taulu and Simola, 2006). Next, continuous data were downsampled to 250 Hz. Physiological artifacts of ocular or cardiac origin were identified via independent component analysis and regressed out of the data. The analysis was performed in sensor space. Twelve planar gradiometers covering the left sensorimotor cortex were selected for the analysis of both resting-state and task data (Fig. 2*A*).

The onset of EMG activity was determined in Go trials. This was defined as the first point after the Go stimulus onset where EMG signal exceeded the critical value (calculated as the mean of prestimulus activity [−1 to 0 s] + 3*SD). The cleaned data were then epoched with respect to the onset of EMG (from −1.5 to + 1.5 s). Any Go trials in which no response was made were rejected. In a final step, data were imported into Fieldtrip and inspected visually using the semiautomatic rejection tool to eliminate any remaining trials with excessive variance in the frequency band of interest (<2% of all trials).

##### Time–frequency analysis of Go/NoGo data.

A time–frequency representation of power was computed using a short-time Fourier transform with a sliding time window of 400 ms that was advanced over the data in 80 ms steps. Power estimates were calculated for frequencies from 2 to 100 Hz in 2 Hz steps. A multitaper method (Percival and Walden, 1993) was used to achieve frequency smoothing of ±4 Hz. Baseline correction was applied by subtracting the mean power values between 0 and −1 s relative to onset of the Go stimulus. The Go trials were averaged and then grand averaged across participants (Fig. 2*C*, left).

Due to the absence of consistent movement-related gamma activity (60–90 Hz range) and a distinct peak at this frequency in more than half of subjects, the grand mean time–frequency representation was plotted and, based on that, 75 Hz was selected as stimulation frequency (Fig. 2*C*, left). The topographies of the MRGS (frequency range: 60–90 Hz) and MRBD (frequency range: 15–30 Hz) for the movement period (time window 0–0.5 s) are shown in Figure 2*C*, right. The temporal and spatial patterns of these oscillatory activities were consistent with previous studies (Cheyne et al., 2008; Gaetz et al., 2011; Cheyne and Ferrari, 2013; Kilavik et al., 2013).

##### Peak detection.

The detection of individual oscillatory peaks in the beta range (15–30 Hz) was performed on cleaned resting-state MEG data using an in-house script. Oscillatory activity was estimated using a fast Fourier transform. Smoothing was achieved using a Savitzky–Golay filter with the order of 3 and window length of 3 Hz. The peak frequencies were detected by first identifying zero-crossings in the differential of each participant's spectrum before linear interpolation of the differential around the zero was used to locate the peak maximum (Fig. 2*B*). Eleven subjects showed a distinct beta band peak ranging from 16 to 23 Hz.

#### Motor learning task

Subjects performed a visually cued RT task after the MEG recording (Session 1). As described previously (Stagg et al., 2011), four horizontal bars were displayed on the screen, each of which corresponded to a key on the keyboard. When a bar changed into an asterisk, subjects were instructed to press the corresponding key as quickly and accurately as possible. The task included sequence blocks consisting of three repeats of a 10-item sequence. The first and 15th blocks consisted of 30 visual cues presented in a random order.

RT was calculated as the time from cue onset to a correct button press. Anticipatory responses, or those that occurred before the cue, were discarded. RTs outside of the mean value ± 2 SD for each block were also excluded. One participant was excluded from analysis due to misunderstanding the instructions.

A motor learning score was calculated for each subject as a percentage change from the RT in the first sequence block to blocks 10–14, when the learning plateaued (Stagg et al., 2011).

#### RT task

At the start of each tACS/TMS session (Sessions 2–4), subjects performed a simple RT task consisting of 20 trials in the absence of TMS to characterize their individual RT. The same RT task was also performed in conjunction with TMS before and after tACS. Subjects were instructed to respond to a visual Go signal (colored green circle) by performing an index finger abduction of the right hand as quickly as possible. Visual stimuli appeared at random intervals (5–7 s) and the subjects were instructed to avoid anticipation of the Go signal and to relax their hand while the fixation cross was displayed on the screen. Stimuli were generated using the MATLAB Psychophysics Toolbox version 3.0 package (Brainard, 1997).

##### RT data analysis.

EMG data acquired during RT task performed in the absence of TMS were analyzed online using Signal software version 3.13 (Cambridge Electronic Design). RT was defined as the time interval (in milliseconds) between the Go signal and the onset of EMG activity in the FDI muscle. The onset of EMG was identified in each trial as the first data point in which the signal exceeded 0.1 mV. The identified RT was then used in the remainder of that experimental session to calculate the timing of the pre-movement TMS pulses.

The data from RT task performed in conjunction with TMS before and after tACS were analyzed offline using MATLAB (The MathWorks). The onset of EMG was identified in each trial as the time when the signal exceeded a threshold of 3 SDs of the EMG activity in the 150 ms period preceding the onset of TMS artifact. RT was defined as the time interval between the Go signal and the onset of EMG activity in the FDI muscle and RTs outside mean ± 2 SD for each block were excluded.

#### tACS

tACS was delivered via a DC stimulator (NeuroConn) through a pair of conductive rubber electrodes (5 × 7 cm^2^). Chloride-free conductive gel was used as a conducting medium between the scalp and the electrodes. One electrode was centered over the TMS-derived FDI hotspot of left M1 (see below); the other was positioned on the contralateral supraorbital ridge.

tACS was administered in a within-subject design. Subjects participated in two active sessions, with different tACS frequency applied at each session, and one sham session. All sessions were separated by at least 1 week. Subjects received tACS at IBF or, if no distinct beta peak was identified, group-averaged beta frequency (mean 20.1 Hz, SD = 2.07), gamma frequency (75 Hz), or sham stimulation.

Stimulation intensity was determined on a subject-by-subject basis and was set to be the highest amplitude that did not elicit phosphenes or discomfort. This resulted in an average stimulation intensity (peak-to-peak) of 0.69 ± 0.11 mA for beta frequency tACS and 1.3 ± 0.36 mA (mean ± SD) for gamma frequency tACS. Current was ramped up and down over the first and last 5 s of stimulation. The total duration of stimulation was 20 min for active sessions and 10 s for the sham session. Impedance was kept at <5 kΩ. Subjects were blinded to the stimulation condition used.

#### TMS

##### Data acquisition.

All TMS data were acquired using a monophasic BiStim machine connected to a 70 mm figure-of-eight coil (Magstim). The left M1 was stimulated in all subjects. The optimal scalp position to elicit MEPs (referred to as the motor hotspot) in the right FDI muscle was determined before each session, with the TMS coil held at 45° to the midsagittal line with the handle pointing posteriorly. The hotspot was marked on a tight-fitting cap to ensure reproducible coil positioning and TMS pulses were delivered through the M1 electrode. For the duration of the sessions, the subjects were seated comfortably in an armchair with their eyes open.

Surface EMG was recorded via disposable neonatal ECG electrodes (Henley's Medical) from the FDI of the right hand using a belly-tendon montage with a ground electrode over the ulnar styloid process. Signals were sampled at 5 kHz, amplified, filtered (10 Hz-1 kHz), and recorded using a CED 1902 amplifier, a CED micro1401 A/D converter, and Signal software version 3.13 (Cambridge Electronic Design).

##### Motor thresholds.

The resting 1 mV motor threshold (MT_1mV_) and active motor threshold (aMT) were determined at the beginning of each tACS/TMS session. The MT_1mV_ was defined as the minimum stimulus intensity required for eliciting an MEP of ∼1 mV peak-to-peak amplitude in at least 5 of 10 consecutive trials in the relaxed FDI muscle. The active motor threshold was defined as the minimum stimulus intensity necessary to evoke a 200 μV peak-to-peak MEP in at least 5 of 10 consecutive trials while subjects maintained ∼30% of the maximum contraction of the FDI.

##### Paired-pulse TMS protocols.

Two paired-pulse protocols were performed in the study: SICI with an interstimulus interval (ISI) of 2.5 ms to assess GABA_A_ synaptic activity (Kujirai et al., 1993; Di Lazzaro et al., 2005) and intracortical facilitation (ICF) with an ISI of 12 ms as a measure of NMDA receptor activity (Ziemann et al., 1996). The conditioning stimulus was set at 70% of aMT and the test stimulus was set at MT_1mV_ for both protocols.

##### Rest measures.

Resting-state MT_1mV_, SICI, and ICF were measured before, during (at T1 = 5 min and at T2 = 15 min after tACS onset), and after tACS. Fifteen trials per condition were applied before and after tACS and 10 trials per condition were applied during tACS. Due to potential changes in motor cortex excitability after tACS, 10 single-pulse MEPs were recorded at the original intensity immediately after stimulation had ceased. If the amplitude of the resulting MEP differed markedly from 1 mV, then the stimulation intensity was altered accordingly (Nitsche et al., 2005; Amadi et al., 2015) before other post-tACS measures were taken. MT_1mV_, SICI, and ICF were delivered in a pseudorandomized order (Fig. 1*B*).

##### Pre-movement measures.

MT_1mV_ and SICI were also measured during the pre-movement period in a simple RT paradigm before and after tACS. To avoid any potential interactions between tACS and task performance, pre-movement measures were not performed during stimulation. The TMS measures were performed in a pseudorandomized order at two different timings during movement preparation: an early time point (25% of mean RT) and a late time point (65% of mean RT), resulting in four different pre-movement protocols: MT_1mV early_, MT_1mV late_, SICI_early_, and SICI_late_. The 25% and 65% RT were adjusted to each subject's mean RT according to a previously described procedure (Murase et al., 2004; Hummel et al., 2009). Fifteen trials per condition and time point were recorded (Fig. 1*B*).

##### MEP data analysis.

Trials were excluded if the test pulse alone failed to elicit a reliable MEP (amplitude <0.1 mV), there was precontraction in the target FDI muscle (EMG amplitude >0.1 mV in the 80 ms preceding the pulse), or, for the pre-movement TMS measures, EMG onset coincided with TMS pulse or no response was made. The peak-to-peak amplitude for each MEP was then calculated. Any MEPs outside of 2 SDs of the mean for each condition for each block were rejected. Next, a single iteration of Grubbs' test with a significance level of 0.05 was performed for each TMS condition separately and any significant outliers excluded. Collectively, these rejection criteria resulted in the exclusion of <5 trials per subject in any condition.

Given that precontraction at very low EMG levels can affect MEP amplitudes, the above exclusion criterion may not be sufficient to detect such subthreshold fluctuations in the signal. To further control for background EMG activity, we calculated the root mean square of the EMG signal in the 80 ms preceding the TMS pulse. A repeated-measures (RM) ANOVA with one factor of session (beta, gamma, sham), one factor of protocol (rest protocols or pre-movement protocols), and one factor of time (baseline, post, or T1, T2) showed no significant main effects or interactions (*p* < 0.05), suggesting that there were no differences in EMG activity between the experimental conditions.

SICI and ICF were expressed as a ratio of the mean conditioned MEP amplitude to the mean unconditioned MEP amplitude (MT_1mV_). For the pre-movement data, the TMS measures were analyzed separately for each pre-movement time point (0.25 and 0.65 RT).

Finally, to investigate the offline effect of tACS on dynamic changes in corticospinal excitability and intracortical inhibition over the pre-movement time span, a linear function was fitted to each subject's MT_1mV_ and SICI amplitudes (expressed as a ratio of the mean conditioned stimulus to the unconditioned stimulus amplitude [MT_1mV_] occurring temporally closest to it) against the position in the pre-movement period when the TMS pulse was delivered. The effect of tACS on the slope of this function was then assessed. The steeper the slope, the higher the increase in dynamic MT_1mV_ and SICI modulation (less inhibition) approaching movement.

#### Statistical analysis

Statistical analyses were performed using MATLAB 8 software (version R2014b; The MathWorks), Prism (version 7; GraphPad Software), and SPSS (version 22.0; IBM).

Normality of data distribution was tested by D'Agostino–Pearson omnibus normality test. All MEP and RT data passed the normality test. Data were analyzed using RM ANOVA. In the case of significant effects, *post hoc* analyses with paired *t* tests (two-tailed) were applied. Mauchly's test was used to test for assumption of sphericity and Greenhouse–Geisser corrections were applied as necessary. Effect size estimates were computed using partial η squared (η2*p*) for RM ANOVAs and Cohen's *d*_z_ for *t* tests. Correlations were assessed using the Spearman's rank correlation method (ρ) and the obtained correlation coefficients were converted into *z*-values with Fisher's *r*-to-*z* transformation. Paired *t* tests (two-tailed) were then used to test for differences between correlations. To prevent a bias, only the subjects for whom the data were available for all sessions were considered for correlational analyses. The significance level for all tests was set at *p* < 0.05.

## Results

We first wished to ensure that there were no systematic differences between the sessions at baseline. For each metric, we performed a RM ANOVA with one factor of session (beta, gamma, sham) on the baseline data. There was no difference in motor thresholds between stimulation sessions (MT_1mV_: *F*_(2,34)_ = 0.564, *p* = 0.574; aMT: *F*_(2,34)_ = 0.398, *p* = 0.675). Further, there were no differences between sessions in MEP amplitude resulting from any of the protocols performed at rest (MT_1mV_: *F*_(2,30)_ = 0.356, *p* = 0.703; SICI: *F*_(2,26)_ = 2.064, *p* = 0.147; ICF: *F*_(2,30)_ = 0.356, *p* = 0.703) or during movement preparation (MT_1mV early_: *F*_(2,30)_ = 0.165, *p* = 0.849; MT_1mV late_: *F*_(2,30)_ = 0.669, *p* = 0.520; SICI_early_: *F*_(2,32)_ = 2.195, *p* = 0.128; SICI_late_: *F*_(2,30)_ = 0.493, *p* = 0.616). Finally, there was no difference in baseline mean RT values between sessions (*F*_(2,30)_ = 0.207, *p* = 0.814).

Next, to confirm that there was no difference between the amplitude of MT_1mV_ at baseline and the amplitude of MT_1mV_ adjusted after stimulation, a RM ANOVA with one factor of session (beta, gamma, sham) and one factor of time (baseline, post) was performed. There was no main effect of time (*F*_(1,14)_ = 0.594, *p* = 0.158) or session (*F*_(2,28)_ = 0.462, *p* = 0.635) and no session × time interaction (*F*_(2,28)_ = 3.182, *p* = 0.057).

Finally, to verify that our paired-pulse protocols elicited the expected inhibition and facilitation at rest, a RM ANOVA with one factor of session (beta, gamma, sham) and one factor of protocol (MT_1mV_ and ppTMS) was performed on the mean baseline values. A significant main effect of protocol was found when MT_1mV_ was compared against SICI (*F*_(1,14)_ = 8.582, *p* = 0.011) and ICF (*F*_(1,15)_ = 12.977, *p* = 0.003). *Post hoc* analyses showed that, as expected, the SICI protocol led to a significant inhibition, and ICF to a significant facilitation, of MEP amplitudes in all sessions.

### Effects of tACS on physiological measures at rest

Having confirmed that there were no systematic differences between sessions and that our TMS protocols had the expected effects, we then wished to examine the effects of driving oscillatory activity on resting cortical physiology.

#### tACS did not affect corticospinal excitability

**Figure 3.**
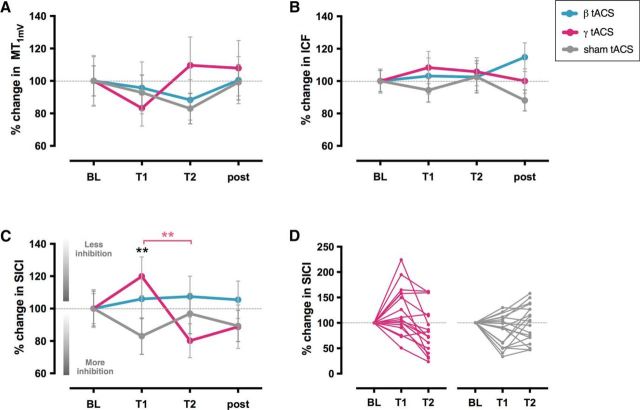
The effect of tACS on cortical excitability measures. Average MEP amplitude (±SEM) values for MT_1mV_ (***A***), ICF (***B***), and SICI (***C***) at baseline (BL), T1 (5 min), and T2 (15 min) and after tACS for all sessions: beta, gamma, and sham. ***D***, Individual subject data showing MEP amplitude values for SICI at BL, T1 and T2 in gamma (left) and sham sessions (right). MEP amplitudes are given relative to baseline (set as 100%). SICI amplitude was increased at T1 by gamma tACS compared with sham session (black asterisks) and compared with T2 in gamma session (pink asterisks). *p < 0.05; **p < 0.005.

We first investigated the effects of driving oscillatory activity on corticospinal excitability, as reflected by a single MEP amplitude (MT_1mV_). A RM ANOVA with one factor of session (beta, gamma, sham) and one factor of time (baseline, T1, T2, post) revealed no significant effect of tACS on corticospinal excitability (no main effect of time [*F*_(3,33)_ = 0.179, *p* = 0.910, η2*p* = 0.016] or session [*F*_(2,22)_ = 2.421, *p* = 0.112, η2*p* = 0.180] and no significant session × time interaction [*F*_(6,66)_ = 0.754, *p* = 0.526, η2*p* = 0.064]; Fig. 3*A*).

#### Gamma tACS modulated GABA_A_ inhibition in a duration-dependent manner during stimulation

We then investigated the effects of tACS on local GABA_A_ inhibition, as reflected by SICI. A RM ANOVA with one factor of session (beta, gamma, sham) and one factor of time (baseline, T1, T2, post) was performed. The analysis revealed no main effect of time (*F*_(3,39)_ = 1.484, *p* = 0.234, η2*p* = 0.102) or session (*F*_(2,26)_ = 0.542, *p* = 0.588, η2*p* = 0.040), but a significant session × time interaction (*F*_(6,78)_ = 3.191, *p* = 0.007, η2*p* = 0.197).

Follow-up RM ANOVAs with one factor of session (active stimulation, sham) and one factor of time (baseline, T1, T2, post) revealed a significant change in SICI in the gamma session compared with sham (no main effect of time [*F*_(3,42)_ = 2.267, *p* = 0.095, η2*p* = 0.139] or session [*F*_(1,14)_ = 0.305, *p* = 0.589, η2*p* = 0.021], but a significant session × time interaction [*F*_(3,42)_ = 5.122, *p* = 0.004, η2*p* = 0.268]). SICI in the gamma session was also significantly different compared with SICI in the beta session (no main effect of time [*F*_(3,39)_ = 1.451, *p* = 0.243, η2*p* = 0.100] or session [*F*_(1,13)_ = 0.831, *p* = 0.379, η2*p* = 0.060], but a significant session × time interaction [*F*_(3,39)_ = 4.107, *p* = 0.013, η2*p* = 0.240]). There was no significant difference between the beta and sham sessions.

*Post hoc t* tests demonstrated a significant decrease in SICI during gamma tACS compared with sham at T1 (*t*_(15)_ = 3.820, *p* = 0.002, Cohen's *d*_z_ = 0.955), but not at T2 (*t*_(16)_ = −1.432, *p* = 0.171, Cohen's *d*_z_ = −0.347) (Fig. 3*C*,*D*). SICI at T1 in the gamma session was also significantly smaller than at baseline (*t*_(15)_ = 2.343, *p* = 0.033, Cohen's *d*_z_ = 0.586) and at T1 in the beta session (*t*_(15)_ = 3.317, *p* = 0.005, Cohen's *d*_z_ = 0.829), whereas SICI in the sham session increased significantly compared with baseline (*t*_(17)_ = −2.437, *p* = 0.026, Cohen's *d*_z_ = −0.574). Within-session analyses revealed that SICI at T1 in the gamma session was significantly smaller than at T2 (*t*_(16)_ = 4.359, *p* < 0.001, Cohen's *d*_z_ = 1.057). In contrast, no difference was observed between SICI at T1 and T2 in the sham session (T2: *t*_(17)_ = −1.959, *p* = 0.067, Cohen's *d*_z_ = −0.462).

#### tACS did not modulate glutamatergic activity

We next investigated the effects of tACS on glutamatergic activity, as reflected by ICF. Consistent with the lack of effect on single-pulse MEPs, which are also thought to partly reflect glutamatergic activity, a RM ANOVA with one factor of session (beta, gamma, sham) and one factor of time (baseline, T1, T2, post) revealed no main effect of time (*F*_(3,42)_ = 0.650, *p* = 0.587, η2*p* = 0.044) or session (*F*_(2,28)_ = 0.035, *p* = 0.966, η2*p* = 0.002) and no significant session × time interaction (*F*_(6,84)_ = 1.598, *p* = 0.158, η2*p* = 0.102) (Fig. 3*B*).

#### Gamma tACS-induced change in GABA_A_ activity was unrelated to stimulation-induced change in corticospinal excitability

Although our results showed that a significant gamma tACS-induced change in GABA_A_ inhibition at T1 was not accompanied by a significant change in corticospinal excitability (a single MEP amplitude), we also investigated whether these two metrics of cortical excitability could be directly linked. We found no correlation between MT_1mV_ and SICI amplitude at T1 (ρ = −0.246, *p* = 0.375) or at T2 (ρ = −0.189, *p* = 0.498).

#### Baseline pre-movement GABA_A_ activity was related to gamma tACS-induced changes in resting GABA_A_ inhibition

Finally, we wished to explore the potential mechanism underlying the gamma tACS-induced decrease in GABA_A_ activity. Specifically, we hypothesized that, if gamma tACS engages endogenous circuits, then the magnitude of the GABA_A_ decrease in response to tACS should be related on a subject-by-subject basis to the magnitude of GABA_A_ release in the pre-movement period close to movement onset, when an endogenous engagement of local interneuronal circuits that are central to the generation and maintenance of gamma oscillations would be expected.

**Figure 4.**
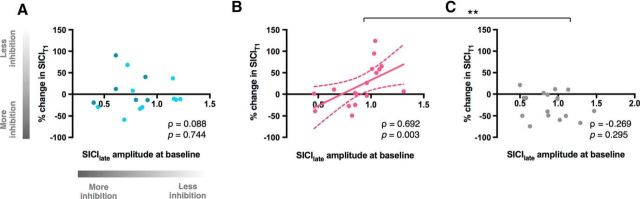
Relationship between pre-movement SICI_late_ before stimulation and percentage change in resting-state SICI at early time point during stimulation (SICI_T1_) relative to baseline in beta tACS (***A***), gamma tACS (***B***), and sham sessions (***C***). Light blue circles in correlations for beta session (***A***) represent participants who received IBF tACS. *p < 0.05; **p < 0.005.

To this end, we performed correlations between pre-movement SICI_late_ at baseline and resting SICI changes recorded during stimulation (at T1 = 5 min and T2 = 15 min) for each session. The results revealed a positive association between baseline pre-movement SICI_late_ and change in SICI_T1_ in gamma session (ρ = 0.692, *p* = 0.003), but not in beta or sham sessions (beta: ρ = 0.088, *p* = 0.744; sham: ρ = −0.269, *p* = 0.295; gamma vs sham: Z = 3.12, *p* = 0.002; beta vs sham: Z = 0.97, *p* = 0.333) (Fig. 4).

#### Gamma tACS-induced modulation of GABA_A_ inhibition predicted an individual's ability to learn a motor task

Finally, given the converging evidence demonstrating the critical role of GABA_A_ inhibition in both motor cortical oscillations and motor learning (Gaetz et al., 2011; Hall et al., 2011; Stagg et al., 2011; Pollok et al., 2014), we set out to determine whether tACS-induced changes in GABA_A_ inhibition were related to individual differences in motor learning performance.

First, we determined whether subjects were able to learn the task. As expected, RT decreased significantly across successive sequence blocks (*F*_(14,140)_ = 9.972; *p* < 0.001). In contrast, there was no significant difference in mean RT between the two random blocks (*t*_(16)_ = 0.548; *p* = 0.591), whereas there was a significant difference between block 14 (the final learning block) and block 15 (the second random block) (*t*_(13)_ = −9.611; *p* < 0.001), suggesting that improvements in RT occurred via learning of a specific sequence and not generic skill learning. There was also no significant difference between the RT from blocks 10–14, which were on the plateau of the learning curve (*F*_(4,48)_ = 0.984; *p* = 0.425).

**Figure 5.**
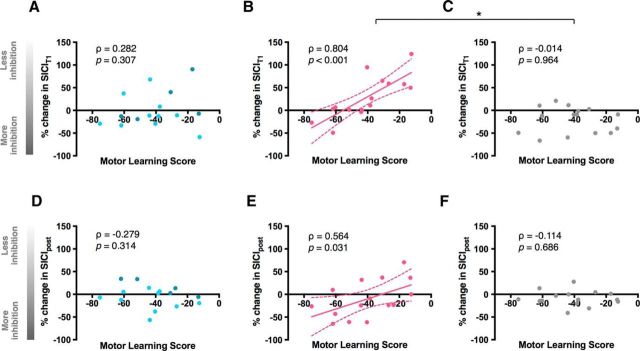
Relationship between motor learning score and stimulation-induced change in SICI at early time point during (SICI_T1_; top) and after stimulation (SICI_post_; bottom) in beta (***A***, ***D***), gamma (***B***, ***E***), and sham sessions (***C***, ***F***). Light blue circles in correlations for beta session (***A***, ***D***) represent participants who received IBF tACS. *p < 0.05; **p < 0.005.

We identified a strong correlation between motor learning performance and the change in SICI_T1_ and, to a lesser extent, a change in SICI_post_ induced by gamma tACS (T1: ρ = 0.804, *p* < 0.001; post: ρ = 0.564, *p* = 0.031) (Fig. 5*B*,*E*). Specifically, we observed that individuals who exhibited a greater increase in GABA_A_ inhibition at an early time point during and after gamma tACS performed better in the motor learning task. No significant association was detected between motor learning and tACS-induced change in SICI in beta (T1: ρ = 0.282, *p* = 0.307; post: ρ = −0.279, *p* = 0.314; Fig. 5*A*,*D*) or sham sessions (T1: ρ = −0.014, *p* = 0.964; post: ρ = −0.114, *p* = 0.686; Fig. 5*C*,*F*). In addition, the correlation between motor learning and gamma tACS-induced change in SICI_T1_ was significantly different compared with sham (Z = 2.76, *p* = 0.006), but not compared with beta (Z = 1.92, *p* = 0.055).

#### Relationship between current intensity and the magnitude of the observed physiological effects

Due to our experimental procedures, a significantly higher peak-to-peak current amplitude was used for gamma tACS compared with beta tACS. We therefore wished to investigate whether the effects seen due to gamma stimulation could be attributed directly to the higher current intensity. First, correlation analysis was performed to assess whether there was a relationship between gamma tACS-affected SICI_T1_ and current intensity. No significant correlation was found between the amplitude of SICI_T1_ and stimulation intensity (ρ = −0.129; *p* = 0.630).

Second, we used a median split procedure in which participants within each active stimulation session were divided into two groups, low and high stimulation intensity. Next, SICI_T1_ measures in the gamma session, low-stimulation group were compared separately against those in the beta session, high-stimulation group. Similarly, SICI_T1_ measures in the gamma session, high-stimulation group were compared against those in the beta session, low-stimulation group. None of the group comparisons performed was significant (*p* > 0.05).

### After-effects of tACS on pre-movement physiological measures

We then tested whether applying tACS for a prolonged period of time had an after-effect on pre-movement corticospinal excitability and GABA_A_ inhibition.

#### tACS had no after-effect on pre-movement cortical excitability

Based on the well recognized distinct patterns of changes in beta- and gamma-frequency power during movement preparation, in parallel to changes in cortical excitability, we wished to investigate whether tACS had any after-effects on pre-movement physiological measures. RM ANOVAs with the factor of session revealed no differences between any of the sessions in pre-movement MT_1mV_ modulation (slope) (*F*_(2,30)_ = 0.695, *p* = 0.507, η2*p* = 0.044) nor in SICI modulation (*F*_(2,26)_ = 2.660, *p* = 0.089, η2*p* = 0.170).

We also investigated whether tACS had any effect on MT_1mV_ or SICI amplitude separately at an early (0.25 RT) or late (0.65 RT) pre-movement time point and found no effect in any of the stimulation sessions (*p* > 0.05).

#### Beta tACS-induced changes in RT were related to tACS-induced changes in corticospinal excitability

Based on the previously proposed antikinetic and prokinetic role of beta and gamma oscillations, respectively (Pogosyan et al., 2009; Joundi et al., 2012), we wished to investigate whether prolonged application of oscillatory currents at beta and gamma frequencies had any after-effect on RT. A RM ANOVA with one factor of session (beta, gamma, sham) and one factor of time (baseline, post) revealed no significant change in RT after stimulation had ceased (no main effect of time [*F*_(1,15)_ = 2.586, *p* = 0.129, η2*p* = 0.147] or session [*F*_(2,30)_ = 0.711, *p* = 0.499, η2*p* = 0.045] and no significant session × time interaction [*F*_(2,30)_ = 0.968, *p* = 0.391, η2*p* = 0.061]).

Given the substantial interindividual variability in behavioral responses after tACS, we aimed to explore the underlying physiological basis of any tACS-associated contribution to the variability of behavioral responses between individuals. We found that beta tACS-induced changes in RT were associated with changes in corticospinal excitability during the late pre-movement period in the beta session (ρ = 0.712, *p* = 0.003), but not in the gamma (ρ = 0.296, *p* = 0.283) or sham (ρ = 0.118, *p* = 0.676) sessions.

## Discussion

This study was performed to investigate the physiological basis and functional significance of driving oscillatory activity in the M1 using tACS. As hypothesized, gamma frequency tACS led to a significant reduction in GABA_A_ inhibition, as assessed by SICI. Interestingly, a reversal of this effect was observed at a later stimulation period, which, to our knowledge, constitutes the first-time evidence of homeostatic changes induced by prolonged application of oscillatory currents. Further, the change in magnitude of GABA_A_ inhibition due to gamma tACS was positively related to the magnitude of GABA_A_ inhibition observed during task-related synchronization of oscillations in inhibitory interneuronal circuits, supporting the hypothesis that tACS engages endogenous oscillatory circuits. Finally, the change in inhibition was strongly related to an individual's ability to learn a motor task.

### Gamma tACS modulated GABA_A_ inhibition during stimulation

We have shown that gamma tACS modulated GABA_A_ inhibition significantly during stimulation. There is broad consensus from the animal and human literature that gamma oscillations reflect underlying GABAergic activity (Towers et al., 2004; Mann and Paulsen, 2007; Gaetz et al., 2011). In agreement with our hypothesis, the exogenous application of a prokinetic gamma rhythm reduced GABA_A_ inhibition early during stimulation. It is worth noting that, although the predominant oscillatory activity in the sensorimotor cortex at rest is at beta frequency (Salmelin and Hari, 1994), different types of neurons differ in the degree to which they are intrinsically responsive to gamma-band synchrony in their inputs (Cardin et al., 2009; Otte et al., 2010). It is therefore conceivable that local inhibitory subnetworks that exhibit gamma frequency oscillations despite being disengaged at rest are more susceptible to the exogenously applied oscillatory current at this frequency because it is closest to their resonant frequency. The changes induced in the affected circuits may manifest as, for example, changes in GABA_A_ inhibition, as shown here.

Despite our initial hypothesis that beta tACS would increase TMS-assessed GABA_A_ inhibition, we found no effect of beta tACS on this measure. It is difficult to know how to interpret this lack of an effect, especially given the extensive animal and simulation literature that strongly suggests a role for GABA_A_ interneurons in beta oscillations (Jensen et al., 2005; Roopun et al., 2006; Yamawaki et al., 2008; Hall et al., 2010). It is possible that beta tACS was delivered at insufficient intensity in the present study (Moliadze et al., 2012; Cancelli et al., 2015). However, it is worth noting that a recent study also showed no effect of beta tACS on SICI when the phase of tACS was not controlled for despite the use of a higher stimulation intensity (Guerra et al., 2016). Future research will focus on establishing optimized stimulation parameters to increase the value of tACS as a tool to study oscillatory activity and also its therapeutic potential.

### Magnitude of tACS-induced changes in GABA_A_ inhibition was related to GABA_A_ change in a period of gamma recruitment

Whether tACS is capable of driving activity in circuits that are not engaged is still an open question. To start to address this point, we investigated the relationship between the physiological changes in response to gamma and beta tACS and the physiological changes (before stimulation) during the pre-movement period, when endogenous gamma activity is increasing and beta activity decreasing. We showed a positive association between the magnitude of gamma tACS-induced change in GABA_A_ inhibition and the magnitude of late pre-movement GABA_A_ activity, supporting the hypothesis that tACS engages local inhibitory circuits involved in the generation of gamma frequency oscillations. In addition, these findings may be of importance for clinical stimulation protocols because they may allow us to probe stimulation efficacy before therapeutic application.

### Gamma tACS-induced modulation of GABA_A_ inhibition predicted motor learning

The next question we sought to address was whether gamma tACS was able to modulate intracortical neuronal circuits in a behaviorally relevant manner. We observed a strong association between gamma tACS-induced change in GABA_A_ inhibition and the degree of motor learning such that subjects who demonstrated a greater increase in GABA_A_ inhibition at an early time point during and after stimulation also showed faster short-term learning. Although these findings provide clear evidence for the behavioral relevance of physiological changes induced by gamma oscillatory current in the motor domain, the question remains as to why stimulation-induced modulation of inhibition is related to performance in the motor learning task. Although speculative at this point, a stronger inhibition due to gamma tACS may reflect a higher inhibitory capacity at an individual level, which has been shown to be related to increased precision in GABA_A_ergic transmission and better manual motor performance (Stinear and Byblow, 2004; Beck et al., 2009; Heise et al., 2013). This is also in agreement with a recent study demonstrating that greater pre-movement inhibition is related to successful performance in the motor learning task (J. Dupont-Hadwen, S. Bestmann, C.J. Stagg, unpublished observations).

### Duration-dependent effects of gamma tACS: evidence for homeostatic plasticity?

One striking aspect of gamma tACS-induced changes in cortical excitability was the opposite pattern of responses early (5 min) as opposed to later (15 min) during stimulation. Although not reported previously in the context of tACS, time-dependent reversal of effects have been documented frequently in studies of homeostatic plasticity in human M1, having been induced by other noninvasive brain stimulation techniques (Siebner et al., 2004; Müller et al., 2007; Nitsche et al., 2007; Batsikadze et al., 2013). For example, continuous theta-burst stimulation applied to the human M1 led to an increase in corticospinal excitability, but the opposite effect was observed when the stimulation duration was doubled (Gentner et al., 2008). It is worth noting that, although the duration-dependent changes reported here appear to have a homeostatic character, they do not outlast stimulation offset. Therefore, further research is needed to investigate the duration dependency of exogenously applied oscillatory currents and their potential therapeutic implications.

### After-effects of tACS on pre-movement physiological measures

Based on the state and frequency dependency of tACS effects (Feurra et al., 2013; Neuling et al., 2013; Santarnecchi et al., 2013) and the well recognized distinct patterns of changes in beta and gamma frequency power as well as cortical excitability during movement preparation (Pfurtscheller and Lopes da Silva, 1999; Reynolds and Ashby, 1999; Zaaroor et al., 2003; Muthukumaraswamy, 2010), here, for the first time, we also evaluated whether externally driving oscillatory activity at these frequencies can lead to plastic changes in cortical excitability, as indexed during movement preparation. We did not find any after-effect of tACS on these measures. It is plausible that any potential tACS-driven event-related physiological changes did not outlast the stimulation period. Nonetheless, the ability to evaluate the effects of exogenously applied oscillatory current on cortical excitability in an event-related paradigm, as shown here, opens the door to new investigations in the motor domain, which are likely to provide important new data on the relationship between oscillatory activity and underlying cortical excitability.

### Limitations

In all sessions, tACS was applied at individualized amplitude, below the individual phosphene and/or discomfort threshold. The phosphene and cutaneous perception threshold was lowest for beta tACS, in agreement with previous studies (Kanai et al., 2008; Chaieb et al., 2011; Turi et al., 2013), which resulted in the use of different stimulation intensities for different frequencies of tACS. We have further reported that, to the best of our knowledge, the observed effects were not related to the intensity, but rather to the frequency of stimulation, which, when at sufficient intensity, was capable of inducing the reported effects. Nonetheless, the use of the same intensity for all stimulation sessions would have been necessary to establish equivocally the frequency specificity of the findings presented here.

In addition, we did not include pre-movement TMS measures during tACS because we wished to avoid any potential interactions between stimulation and movement. However, this may have prevented us from demonstrating the relationship between oscillatory activity and the underlying cortical excitability changes that accompany the movement preparation period.

Finally, in approximately half of the participants, the frequency of beta tACS was matched to their individual peak at beta frequency based on the MEG data acquired in Session 1. However, we cannot exclude the possibility of small intraindividual variations in the beta-band peak across sessions.

### Conclusions

Our findings demonstrate that driving gamma frequency oscillations using tACS leads to significant, duration-dependent changes in GABA_A_ inhibition. We also show for the first time a clear relationship between the change in magnitude of GABA_A_ inhibition induced by stimulation and the magnitude of GABA_A_ inhibition observed during task-related synchronization of oscillations in inhibitory interneuronal circuits, supporting the hypothesis that tACS engages endogenous oscillatory circuits. Further, gamma tACS-induced change in inhibition was closely related to an individual's ability to learn a motor task. The findings presented here contribute to our understanding of the neurophysiological basis of motor rhythms and suggest that tACS modulates local endogenous circuits in a behaviorally relevant manner, offering the possibility of developing tACS as a potential therapeutic tool.
